# Water-carbon dioxide solid phase equilibria at pressures above 4 GPa

**DOI:** 10.1038/s41598-017-00915-0

**Published:** 2017-04-11

**Authors:** E. H. Abramson, O. Bollengier, J. M. Brown

**Affiliations:** Department of Earth and Space Sciences, University of Washington, Seattle, WA 98195-1310 USA

## Abstract

A solid phase in the mixed water-carbon dioxide system, previously identified as carbonic acid, was observed in the high-pressure diamond-anvil cell. The pressure-temperature paths of both its melting and peritectic curves were measured, beginning at 4.4 GPa and 165 °C (where it exists in a quadruple equilibrium, together with an aqueous fluid and the ices H_2_O(VII) and CO_2_(I)) and proceeding to higher pressures and temperatures. Single-crystal X-ray diffraction revealed a triclinic crystal with unit cell parameters (at 6.5 GPa and 20 °C) of *a* = 5.88 Å, *b* = 6.59 Å, *c* = 6.99 Å, *α* = 88.7°, *β* = 79.7°, and *γ* = 67.7°. Raman spectra exhibit a major line at ~1080 cm^−1^ and lattice modes below 300 cm^−1^.

## Introduction

The binary water-carbon dioxide system is of broad interest, ubiquitous in Earth and planetary sciences, in industrial chemistry and in life sciences, and has been the subject of extensive experimental and theoretical attention. Mixed solid phases known from experiments include the sI clathrate hydrate which is stable at temperatures below 21 °C and pressures below 0.7 GPa, and a filled ice observed at pressures up to ~1 GPa, above which it decomposes into H_2_O and CO_2_ ices^1–3^.

The 1:1 adduct, carbonic acid (H_2_CO_3_), has been described in the gas phase, rare-gas -matrix studies and aqueous solution (see, for example, references in Mitterdorfer *et al*.^4^ and in Reisenauer *et al*.^5^). The creation of amorphous solid and crystalline forms has required exotic methods of synthesis including the bombardment of cryogenic mixed ices with ionizing radiation (VUV, protons, electrons), implantation of protons into solid CO_2_, and cryogenic preparation of inter-layered deposits of aqueous bicarbonate and acid solutions followed by warming to ~−50 °C. No experiment has yet confirmed whether any of several proposed crystalline structures, based on density-functional-theory calculations^6, 7^, is correct. These solids, with vapor pressures much lower than either carbon dioxide or water, are expected to be produced by interaction of the solar wind with icy moons and comets, thus carbonic acid has been proposed as both a repository of carbonic volatiles and an important intermediate in the carbon cycle of these bodies^8, 9^.

Recently, Wang *et al*. presented evidence of a new solid phase produced in the high-pressure, diamond-anvil cell^10^. Above 2.4 GPa, laser heating of mixtures of H_2_O and CO_2_ resulted in an apparent optical darkening and the production of new IR and Raman bands. The phase was identified as β-H_2_CO_3_ on the basis of spectra similar to those of the cryogenically prepared, 1 bar solid (the previously suggested α-H_2_CO_3_ has been shown to be a methyl ester of the acid^5^). Its domain of thermodynamic stability, however, remained elusive. Over the entire 2.4–4.6 GPa pressure range on which Wang *et al*. synthesized the new phase, only crystallites could be formed and would disappear irreversibly in the presence of the aqueous fluid. Consequently, Wang *et al*. concluded that the new phase was metastable and did not coexist in equilibrium with the fluid.

Here we show that at 4.4 GPa and 165 °C the new phase can be brought to a point of quadruple equilibrium with the aqueous fluid and the ices H_2_O(VII) and CO_2_(I). Equilibrium between the new phase (hereafter designated as S3) and the fluid persists to higher pressures and temperatures, where S3 is readily crystallized from solution. For CO_2_-rich mixtures, S3 melts incongruently, decomposing into CO_2_(I) and an aqueous fluid; conversely, a eutectic system with H_2_O(VII) exists for more water-rich compositions. We report the low-pressure end of the phase diagram of this new solid, along with further Raman spectra extending to the lattice modes. The first, single-crystal X-ray diffraction patterns acquired from several euhedral crystals reveal a triclinic system with a unit cell volume of 245 Å^3^. Presuming S3 to be the suggested anhydrous H_2_CO_3_, agreement with buoyancy observations would require at least 5 formula units in the cell. Raman spectroscopy of the aqueous solution at similar conditions also confirms a change of speciation from CO_2_(*aq*), possibly to H_2_CO_3_(*aq*).

It appears from the present results that in concentrated carbonic solutions H_2_CO_3_(*aq*) might exist under a range of pressures and temperatures characteristic of normal subduction conditions within Earth, while the solid could exist in colder systems or possibly in the interiors of outer icy worlds. This suggests an important role for H_2_CO_3_ in planetary processes, as both an aqueous species and an ice.

## Methods

Water was loaded along with a bubble of air into a modified Merrill-Bassett diamond-anvil cell (DAC). After sealing, the DAC was subsequently immersed in liquid carbon dioxide at 10 °C and 58 bar, briefly re-opened to let CO_2_ displace the bubble, and re-sealed. Gaskets were made of rhenium.

The DAC was placed in an oven with windows in front and back. Temperatures were measured to 1 °C with type K thermocouples located next to the diamonds. Pressure was determined from the Raman-scattered light from one or more pieces of cubic boron nitride (cBN) included in the load. Scattering was induced by 20 mW of 488 nm laser light, focused through the front window by a microscope objective of 0.28 numerical aperture, back-scattered radiation being collected through the same objective. The light was sent to a 0.3 m monochromator with an 1800 lines/mm grating and dispersed onto a CCD camera. To maintain the precision of the pressure measurements, the grating of the monochromator used for this purpose was kept at a fixed position. The frequency, *v*, of the cBN transverse optical phonon was assumed to vary with temperature and pressure as^11, 12^:

$$\nu ={c}_{1}P+{c}_{2}PT+{c}_{3}{P}^{2}+{c}_{4}T+{c}_{5}{T}^{2}+{c}_{6}$$

with *c*_1_ = 3.303 cm^−1^ · GPa^−1^, *c*_2_ = 1.85 × 10^−4^ cm^−1^ · GPa^−1^ · K^−1^, *c*_3_ = −9.72 × 10^−3^ cm^−1^ · GPa^−2^, *c*_4_ = 5.28 × 10^−3^ cm^−1^ · K^−1^, *c*_5_ = −2.94 × 10^−5^ cm^−1^ · K^−2^, and *c*_6_ = 1054.96 cm^−1^.

The precision of pressure measurements was usually 0.1 GPa although, for certain pressures and temperatures, the cBN line was partially overlapped by the (weaker) sample lines seen at 1040 cm^−1^ (Fig. 5) and the precision thereby degraded. Since this occurred only infrequently, for thick loads coupled with smaller pieces of cBN, these data could be discarded.

When Raman spectra of the water-carbon dioxide mixture were to be recorded a (100% reflective) pick-off mirror was inserted into the path of the collected light so as to direct the Raman scattering into a second monochromator (0.25 m, 1200 lines/mm). The slits of this monochromator were set to give a resolution of 5 cm^−1^ (full width at half maximum). The time required to accumulate a suitable spectrum was typically 10–20 minutes.

X-ray diffraction data were taken on line 12.2.2 of the Advanced Light Source (Berkeley) with a monochromatic beam of 25 keV. The beam had a cross-section of 20 μm × 20 μm, allowing it to be focused on single crystals previously identified as the S3 phase through Raman spectroscopy. The diamond supports allowed scattering to be observed over a circular aperture of ±35° around the center-line of the DAC. Data were taken at room temperature.

**Figure 1:**
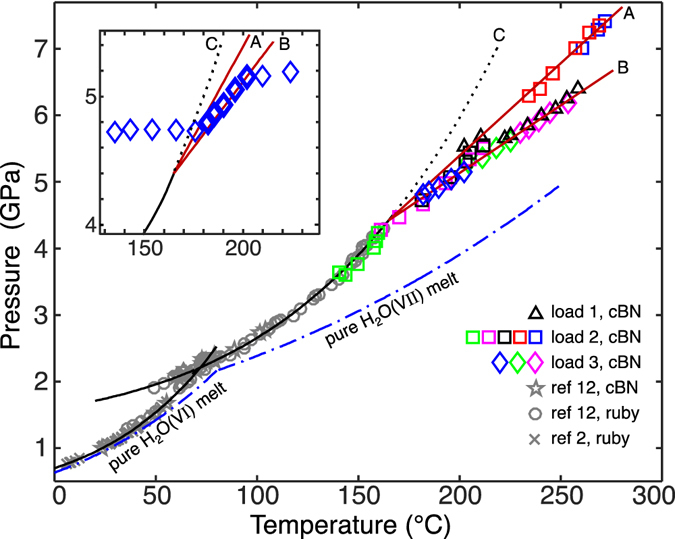
Phase transition points acquired along univariant, 3-phase equilibria in the water-carbon dioxide system. Up to 4.4 GPa, the melting curves of known water ices, VI and VII, in a CO_2_ -saturated fluid (solid black lines) depart progressively from those observed in the pure water system (dash and dot blue lines). Above 4.4 GPa, two new lines diverge from the expected (line C) H_2_O(VII) melting curve, with line A demarking the S3-H_2_O(VII)-fluid equilibrium and line B the S3-CO_2_(I)-fluid equilibrium. Shown in the inset is the pressure-temperature history (with increasing temperature) of a single run taken with load 3. The two, abrupt changes in slope of the trace are indicative of the beginning and the end of the phase transition. The five diamonds drawn with thicker lines indicate points taken during the transition and correspond to the same five symbols in the main body of the figure; all points in the main figure were identified in this manner. At the highest pressures of load 1 (triangles), upon completion of the phase transition CO_2_ ice was seen to remain, while for load 2 (squares) water ice remained.

Phase transitions were determined by first bringing the DAC to a desired starting pressure, then slowly raising the temperature while monitoring changes in pressure. As is usual in the quasi-isochoric DAC, the initiation or completion of a phase transition causes an observable discontinuity in the slope of the pressure-temperature trace (inset, Fig. 1). After completion of a transition, the pressure could be increased and another run performed with the same load. In Fig. 1, points of phase transition observed over several different runs, and three different DAC loads, are collected in a single plot along with lower pressure data from two previous studies.

## Results and Discussion

**Figure 2:**
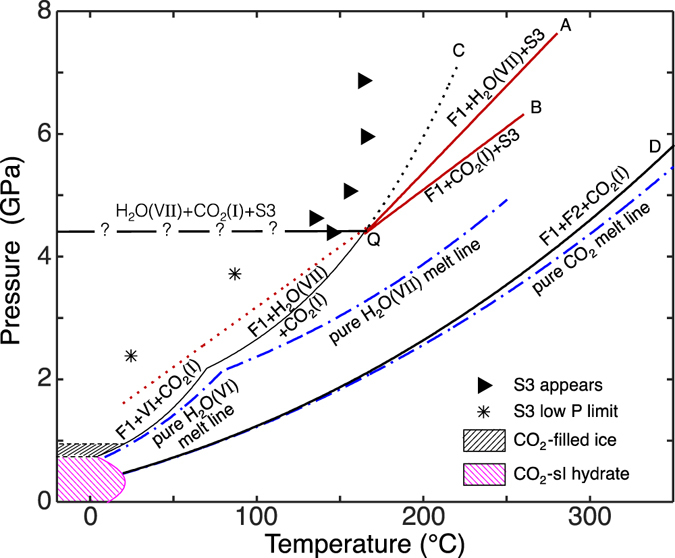
Phase boundaries of the water-carbon dioxide binary. Below 1 GPa, two CO_2_ hydrates (a sI clathrate and a filled ice) have been reported^1–3^. Filled triangles demark where the new phase (S3) has been observed to nucleate upon heating. Asterisks indicate pressures below which S3 was seen to decompose. The black dotted line indicates the (unobserved) metastable extension of the CO_2_-saturated H_2_O(VII) solidus to higher temperatures in the absence of S3, and the red dotted line an approximate metastable extension of the solidus of S3 to lower temperatures. Curves labeled “A” through “D” correspond to the points^12^ (one peritectic, two eutectics, and the melting point) presented in Fig. 3. F1 is an aqueous solution, while melting of CO_2_ produces a fluid, F2, dominated by that compound. The invariant quadruple point, “Q” (H_2_O(VII) + CO_2_(I) + S3 + F1), lies at 4.4 GPa and 165 °C.

**Figure 3:**
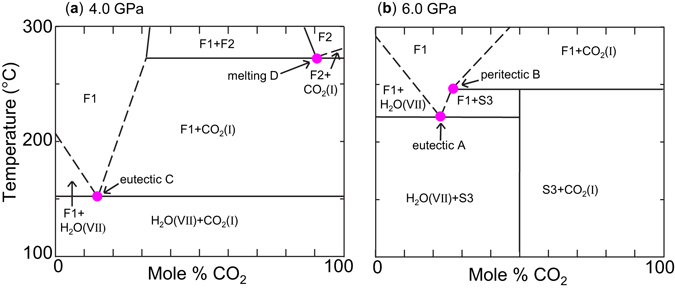
Temperature-composition phase diagrams at 4 and 6 GPa. In the current experiments, temperatures and pressures were measured, however composition values are unknown for most of the diagram, and boundaries indicated by dashed lines are approximate. The two eutectic points (A and C), the peritectic point (B) and the melting point (D) correspond to the similarly designated curves in Fig. 2.

While following the solidus curve of H_2_O(VII) to higher pressures (line C in Figs 1 and 2)^12^, an abrupt change in its slope at 4.4 GPa (line A) and the simultaneous appearance of another univariant line of solid-solid-liquid equilibrium (line B) indicated the presence of a new phase. The Raman spectrum of this solid matches that of the phase reported by Wang *et al*. Solely from the presence of the two new lines of equilibria we can infer that the new solid phase is a composite of both water and carbon dioxide. The lower temperature line (line A) marks the melting of H_2_O(VII) (when water is present in excess of the stoichiometry of production of S3), while the higher temperature line (line B) marks where S3 decomposes (and possibly dissolves into the fluid), in equilibrium with solid CO_2_. Temperature-composition plots of these phase boundaries are shown in Fig. 3, with the four univariant (three phase) lines A–D appearing as points at the selected pressures.

**Figure 4:**
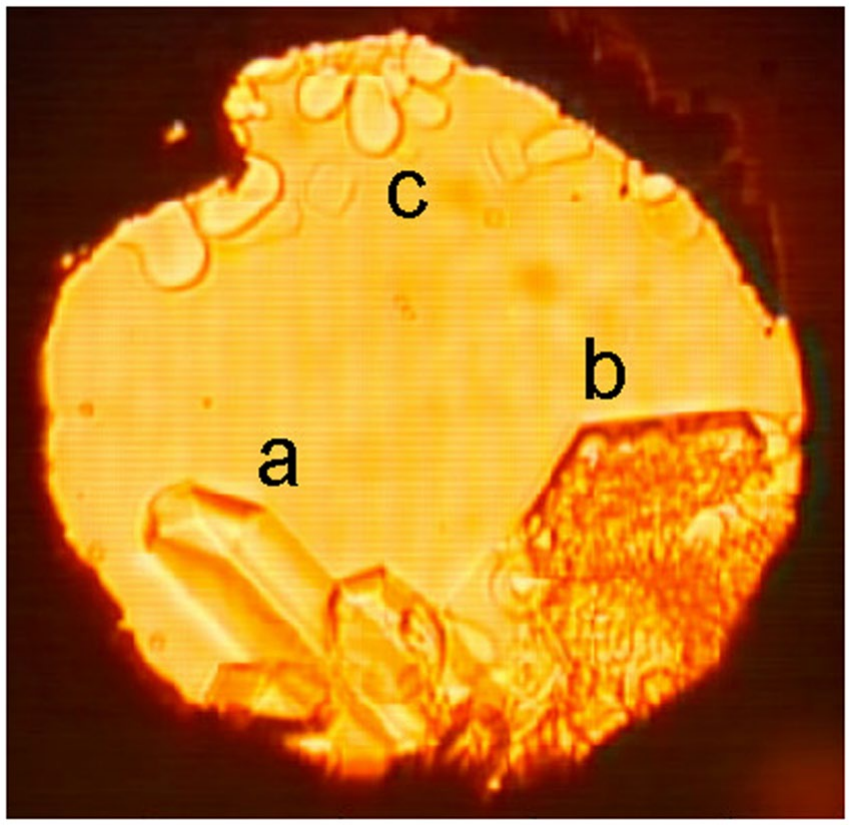
Photomicrograph of crystals growing from a water-CO_2_ fluid at 240 °C and 6.5 GPa. Crystals “a” and “b” are both of phase S3 and were observed to fall through the fluid. At first “b” was clear, but then abruptly darkened, presumably as trapped fluid (non-stoichiometric relative to S3) precipitated crystallites of the H_2_O(VII)-S3 eutectic. Crystals “c” are H_2_O(VII) which floated up through the fluid.

A photomicrograph of crystals of the new phase, grown out of a water-rich fluid along curve “A”, is presented in Fig. 4. In the course of this run, both crystals “a” and “b” were at first clear; crystal “b” subsequently darkened in patches, presumably by trapping a solution which precipitated a micro-crystalline aggregate of the H_2_O(VII)-S3 eutectic. Allowing such a “darkened” crystal to sit for a few hours leads to lightening along the rims which are in contact with solution, as dissolution and re-growth take place. In our experiments, euhedral crystals of S3 could be grown from the fluid phase repeatedly and reversibly. In contrast to the report of Wang *et al*., our observations clearly extend into the regime of thermodynamic stability of the new phase.

Single-crystal X-ray diffraction of S3 revealed a triclinic system with unit cell parameters (at 6.5 GPa and 20 °C) of *a* = 5.88 Å, *b* = 6.59 Å, *c* = 6.99 Å, *α* = 88.7°, *β* = 79.7°, *γ* = 67.7°, and a volume of 246.5 Å^3^. Note that possible crystal structures resulting from the calculations of refs 6 and 7 are all orthorhombic or monoclinic. So far, it has not been possible to experimentally determine the exact crystal structure. In samples with large fractions of melt, S3 crystals could be seen to sink, while H_2_O(VII) crystals rose. For S3 to be denser than H_2_O(VII) at the conditions of growth (1.72 g·cm^−3^)^13^, and assuming its formula is indeed H_2_CO_3_, there must be at least 5 molecules per unit cell for a density of 2.10 g·cm^−3^. If the crystal consists of pairs of hydrogen-bonded molecules, then an even number per unit cell would suggest six molecules for a density of 2.52 g·cm^−3^.

In our experiments, S3 was initially noticed when raising the temperature caused the solid contents of the cell to turn black in transmitted light. In reflected light, however, the contents were seen to be white, indicating the cause of the opacity to be the production of many small crystals rather than the absorption of light; this is in agreement with the observations of Wang *et al*. The transition between S3 and a solid mixture of water and CO_2_ is sluggish, preventing a clear determination of the position of the equilibrium boundary; the dashed line in Fig. 2 is a reminder that such a boundary must exist, connecting to the quadruple point, Q. When heating at pressures above 4 GPa, S3 can be seen to grow in as temperatures reach roughly 150–160 °C (filled triangles in Fig. 2); however, this is clearly due to having surpassed a kinetic barrier, as the reaction is not reversible (subsequent reduction of the temperature to 20 °C does not result in the disappearance of S3). Wang *et al*. report that S3 decomposes at room temperature somewhat below 2.4 GPa, and we have similarly observed decomposition to occur at 85 °C between 3.8 and 3.3 GPa. This, again, may be the result of overcoming a kinetic barrier, as the metastable extension of curve B (Fig. 2) is approached and production of fluid (with immediate freezing into the solid phases of water and CO_2_) becomes possible.

**Figure 5:**
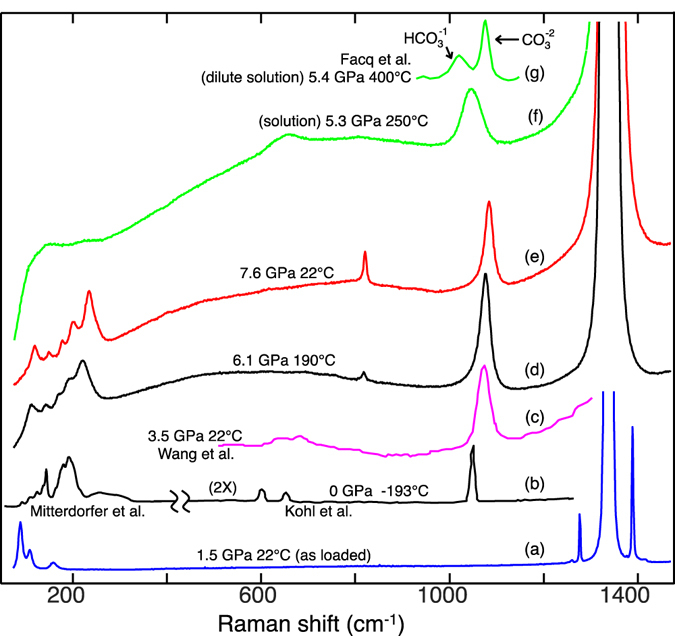
Raman spectra. (a) DAC contents as loaded in our experiments (before warming) show a strong signal which matches that known^14^ for CO_2_(I). In this and subsequent spectra, the truncated peaks at ~1340 cm^−1^ are from the diamonds. (b) Carbonic acid produced cryogenically^4, 15^. (c) New solid phase (S3) reported by Wang *et al*. (d,e) New solid phase (S3) from this study. The sharp mode at ~1080 cm^−1^ is characteristic of the phase. Lattice modes are also prominent. (f) In solution, a line appears at ~1040 cm^−1^, shifted ~40 cm^−1^ to the red and about twice as broad as the nearby S3 line; this may be associated with dissolved molecules of the S3 phase, but may otherwise be due to the bicarbonate ion. (g) Lines from bicarbonate and carbonate ions in a dilute aqueous solution^16^.

When initially loaded (before heating for the first time) our samples produced Raman signals (Fig. 5, trace a) which matched exactly those known for CO_2_(I)^14^, with no other lines in the monitored range below 1500 cm^−1^. The appearance of the distinctive S3 Raman features (described below) is associated with a decrease in the intensity of the solid CO_2_ contributions (up to a complete disappearance for water-rich loads). Decomposition of S3 (when pressure was lowered) was evidenced by a disappearance of the S3 features and a reappearance of those of solid CO_2_.

The Raman spectrum of the new phase is distinguished by a strong, characteristic line located at 1084 cm^−1^ (at 7.6 GPa), with a pressure derivative of ~5 cm^−1^ · GPa^−1^ (Fig. 5, traces d and e), and a weaker line at ~820 cm^−1^. Strong Raman scattering also occurs below 300 cm^−1^, presumably from lattice modes. The relative intensities of the lines vary in strength with the orientation of the crystal probed. As noted by Wang *et al*., the spectrum bears a reasonable resemblance to that of carbonic acid produced as a cryogenic surface deposit (trace b)^4, 15^. The weaker line at ~820 cm^−1^, visible in the current crystal spectra, was not observed by Wang *et al*. (trace c) and, conversely, two small peaks at 640 and 680 cm^−1^, previously noted, were not observed from our samples, perhaps due to the orientations of the crystals. Lines shift systematically with pressure but were not affected significantly by differences in temperature over the range explored in our experiments.

Also shown in Fig. 5 is a spectrum from the fluid phase (trace f). A line appears at 1040 cm^−1^, ~40 cm^−1^ red-shifted from the strong S3 line and broadened, as well as a weaker line at ~650 cm^−1^. While it is tempting to consider these to be from dissolved molecules of the same species forming S3, it is also possible that at least the stronger of the two should be ascribed to the bicarbonate ion. A spectrum containing lines of both bicarbonate and carbonate^16^ in a dilute aqueous solution is shown in trace (g); the spectral shift from the line in (f) may be due to the high concentration of CO_2_ (~30 mole%) in the latter solution.

Should S3 be crystalline H_2_CO_3_, as suggested by Wang *et al*., our observations would then establish for this compound a regime of high-pressure thermodynamic stability, as predicted by simulations^6, 7^. Furthermore, the wide pressure range under which S3 was observed by Wang *et al*., up to 25 GPa, might now also be presumed to represent equilibrium conditions, in agreement with simulations (Saleh and Oganov predicted H_2_CO_3_ to be stable as a solid between roughly 1 and 44 GPa at 27 °C, with a subsequent polymorph stable up to at least 400 GPa). It should be borne in mind that none of the simulated structures matches the triclinic system of our samples; however, discovery of the true crystal structure within the reportedly complex energy landscape of H_2_CO_3_^7^ may have been prevented by small errors in calculation. As well, the calculations of Saleh and Oganov at pressures less than 10 GPa used only 4 formula units of H_2_CO_3_, which, as shown by the combination of our x-ray and density data, is less than the number required for a primitive unit cell.

## Conclusion

Experiments on mixed H_2_O-CO_2_ samples up to 7.6 GPa evidence the existence of a new solid phase (S3). Above a quadruple point at 4.4 GPa and 165 °C, the new phase can be observed along both its melting and peritectic curves, distinct from the known melting curves of H_2_O and CO_2_ ices at the same pressures; it is found to be in equilibrium with the fluid up to the highest investigated pressure. Raman spectra of S3 exhibit a strong line at ~1080 cm^−1^ and weaker one at ~820 cm^−1^, in addition to a complex of lattice modes below 300 cm^−1^. In the fluid, close to the melting curve of S3, another strong line appears at ~1040 cm^−1^; whether this is properly attributed to dissolved molecules of S3, corresponding to the 1080 cm^−1^ line of the solid, or rather to the bicarbonate ion, is not yet clear.

S3 crystallizes in a triclinic system with unit cell parameters (at 6.5 GPa and 20 °C) of *a* = 5.88 Å, *b* = 6.59 Å, *c* = 6.99 Å, *α* = 88.7°, *β* = 79.7° and *γ* = 67.7°. The optical behaviour, spectroscopic features and range of occurrence of S3 are consistent with those reported recently by Wang *et al*. for a new solid phase observed at room temperature between 2.4 and 25 GPa and tentatively identified as crystalline carbonic acid. While these experimental observations are consistent with calculations suggesting that solid H_2_CO_3_ is thermodynamically stable at pressures of several to tens of GPa, the observed triclinic form was not predicted.

Our updated picture of the H_2_O-CO_2_ system reveals an intricate succession of phase transitions likely to govern the behaviour of these species in the crust of terrestrial planets and the hydrosphere of icy worlds. Below 1 GPa, and at temperatures less than 21 °C, H_2_O and CO_2_ associate preferentially as hydrates, with mutual fluid solubilities not exceeding a few percent. Above 4.4 GPa, and at higher temperatures, lies a domain of stability of the putative H_2_CO_3_, in a regime of high solubilities and evolving speciation. H_2_CO_3_ may be stable at pressure to at least tens of GPa, covering a wide range of conditions in planetary interiors. Between 1 and 4.4 GPa, only pure H_2_O and CO_2_ ices have been confirmed as thermodynamically stable solids, and the speciation and miscibility behaviour of the binary system remain largely unknown. Experimental investigation of this behaviour will be necessary to assess geochemical processes involving these two major planetary volatiles.
